# SFRP1 increases TMPRSS2-ERG expression promoting neoplastic features in prostate cancer in vitro and in vivo

**DOI:** 10.1186/s12935-020-01333-5

**Published:** 2020-07-16

**Authors:** Carlos D. Cruz-Hernández, Marian Cruz-Burgos, Sergio A. Cortés-Ramírez, Alberto Losada-García, Ignacio Camacho-Arroyo, Patricia García-López, Elizabeth Langley, Vanessa González-Covarrubias, Monserrat Llaguno-Munive, Martha E. Albino-Sánchez, José L. Cruz-Colín, Carlos Pérez-Plasencia, Fredy O. Beltrán-Anaya, Mauricio Rodríguez-Dorantes

**Affiliations:** 1 Instituto Nacional de Medicina Genómica, Périferico Sur 4809, Arenal Tepepan, 14610 Mexico city, Mexico; 2 Unidad de Investigación en Reproducción Humana, Instituto Nacional de Perinatología-Facultad de Química, Universidad Nacional Autónoma de México; (UNAM), 04510 Mexico City, Mexico; 3 Instituto Nacional de Cancerología, 14080 Mexico city, Mexico; 4 Departamento de Biología celular, CINVESTAV, Av Instituto Politécnico Nacional 2508, San Pedro Zacatenco, 07360 Mexico city, Mexico

**Keywords:** SFRP1, TMPRSS2-ERG, Xenograft

## Abstract

**Background:**

Prostate cancer (PCa) is the second cause of cancer related death in North American men. Androgens play an important role in its progression by regulating the expression of several genes including fusion ones that results from structural chromosome rearrangements. *TMPRSS2*-*ERG* is a fusion gene commonly observed in over 50% of PCa tumors, and its expression can be transcriptionally regulated by the androgen receptor (AR) given its androgen responsive elements. *TMPRSS2*-*ERG* could be involved in epithelial–mesenchymal transition (EMT) during tumor development. ERG has been reported as a key transcriptional factor in the AR-ERG-WNT network where five SFRP proteins, structurally similar to WNT ligands and considered to be WNT pathway antagonists, can regulate signaling in the extracellular space  by binding to WNT proteins or Frizzled receptors. It has been shown that over-expression of SFRP1 protein can regulate the transcriptional activity of AR and inhibits the formation of colonies in LNCaP cells. However, the effect of SFRP1 has been controversial since differential effects have been observed depending on its concentration and tissue location. In this study, we explored the role of exogenous SFRP1 protein in cells expressing the TMPRSS2-ERG fusion.

**Methods:**

To evaluate the effect of exogenous SFRP1 protein on PCa cells expressing TMPRSS2-ERG, we performed in silico analysis from TCGA cohort, expression assays by RT-qPCR and Western blot, cell viability and cell cycle measurements by cytometry, migration and invasion assays by xCELLigance system and murine xenografts.

**Results:**

We demonstrated that SFRP1 protein increased ERG expression by promoting cellular migration in vitro and increasing tumor growth in vivo in PCa cells with the TMPRSS2-ERG fusion.

**Conclusions:**

These results suggest the possible role of exogenous SFRP1 protein as a modulator of AR-ERG-WNT signaling network in cells positive to TMPRSS2-ERG. Further, investigation is needed to determine if SFRP1 protein could be a target in against this type of PCa.

## Background

Prostate cancer (PCa) is the second cause of cancer-related deaths in North American men [1]. The PCa 5-year survival is nearly 100% when is diagnosed at local stage but poor (32.6%) when it’s diagnosed with metastases. Initial therapy consist to blockade of AR activity but it´s effective in short time because PCa progresses to Castration Resistant Prostate Cancer (CRPC) [2–4]. Androgens have an essential role in PCa progression regulating the expression of several genes, including fusion genes [5, 6]. These fusions can result from structural rearrangements, such as deletions and translocations, transcription read-through of neighboring genes or the trans- and cis-splicing of pre-mRNAs [7]. In PCa, *TMPRSS2*-*ERG* is found at high frequency in prostate cancer and it’s over-expressed near to 50% of tumors [8–10]. The expression of this fusion it can be regulated by the transcriptional activity of AR because *TMPRSS2* have androgen responsive elements (ARE’s) as other genes as *KLK2* and *KLK3* [11, 12]. Functional part of *TMPRSS2*-*ERG* is ERG, ERG belongs to the family of ETS transcriptional factors and has been reported that regulates the aberrant expression of WT3A, LEF-1 and FZD4 in PCa cells [13, 14].

It is still debated if only the presence of TMPRSS2-ERG is required for the disease to progress to a more advanced state in PCa. Some reports show that TMPRS2-ERG fusion requires additional molecular events such as a *PTEN* deletion and up-expression of FZD4 to promote PCa progression [15–18]. In this line, ERG has been reported as key transcriptional factor in the AR-ERG-WNT network, however, the role of WNT-related proteins as WIF-1, Cerberus, and SFRP proteins remains to be clarified [19]. SFRP proteins are group of five proteins that are structurally similar to WNT ligands. SFRP proteins are considered to be WNT pathway antagonists, modulating signaling either by binding to WNT proteins or Frizzled receptors but it’s role only as antagonist keep controversial. For example, Kawano et al. reported that SFRP1 protein decreases transcriptional activity of AR in 22rv1 and LNCaP cells through a mechanism independent of beta-catenin [20, 21]. The expression of SFRP1 is low in many models of prostate cancer cells, has been proposed this due a methylation-independent mechanism and even it has been proposed SFRP1 as a possible biomarker in the diagnosis of PCa [22–24]. Despite all that reports, the role of SFRP1 in PCa it´s still controversial, some studies show that SFRP1 it’s over-expressed in stroma and promotes PCa progression participating through the stromal-epithelium communication [25–27]. In addition, as far as we know, it has not been evaluated before, if a possible AR’s modulator such as SFRP1 could also affect TMPRSS2-ERG expression. Here we aimed to evaluate the effect of exogenous SFRP1 protein on the TMPRSS2-ERG expression and the impact over neoplastic features of PCa cells and castrated xenograft model to clarify if this protein could be related to the progression of PCa positive to TMPRSS2-ERG.

## Methods

### Cell culture

LNCaP, VCaP and PC3 cells were purchased from ATCC (Manassas, United States). Cells were grown in RPMI 1640 medium (Sigma-Aldrich, St. Louis, MO, USA) supplemented with fetal bovine serum (FBS) (BioWest, South American origin), at 10% in 37 °C and 5% CO_2_ atmosphere. RWPE-1 cell line was acquired from ATCC and was cultured in Keratinocyte Serum Free Medium (Lonza, Allendale, NJ, USA), PrSC cell line was purchased from LONZA and was cultured in Stromal Cell Basal Medium (LONZA). When cells were treated with DHT hormone, RPMI medium without phenol red (Sigma) supplemented with 5% of charcoal stripped FBS (LONZA) was used.

### Treatments

We performed treatments with SFRP1 protein (SIGMA) at 0.01 nM with 0.1% of BSA carrier buffer as vehicle. DHT at 0.01 nM (SIGMA) was diluted in 0.1% biology grade ethanol as vehicle. The vehicle in control conditions was considered as 0.1% of BSA and 0.1% of ethanol v/v in RPMI medium.

### RT-qPCR

Cells were plated in 25 cm^2^-angled flasks at 1 × 10^6^ cells of confluence, except VCaP cells at 1.5 × 10^6^ cells. Twenty-four hours after treatments, cells were scrapped from flasks for extracting RNA and proteins. RNA extraction was performed with RNAeasy kit (QUIAGEN, Hilden Germany) according to manufacturer’s instructions. Next, cDNA was obtained by retro-transcription assay using Revert Aid Synthesis Kit (Thermofisher, USA). For RT-PCR assays, taqman probes used for were: GAPDH (Hs02758991_g1), KLK3 (Hs02576345_m1), AR (Hs00171172_m1), TMPRSS2 (Hs03063375_ft), ERG (Hs01554631_m1), SFRP1 (Hs00610060_m1) and LEF1 (Hs01547250_m1) purchased from Thermofisher (Massachusetts, USA).

### Western blotting

Protein extraction was performed with RIPA buffer (SIGMA). Proteins were quantified with EZQ chemiluminescent system (BioRad, California, USA). Western blotting assays were performed following the canonical steps except for slight modifications. Proteins were run on 12% polyacrylamide gels and electrophoresis was carried out at 100 V during 1.5 h. A semi-dry system was used for transference to PVDF membrane at 15 V for 30 min. Next, we blocked the membrane with milk at 5% for 2 h. After washes, primary antibodies were added in 1% milk overnight in a shaker. Next day, the membrane was incubated with secondary antibodies in 1% milk for 2 h. Membrane photos were taken after incubation with HRP system Luminata Forte (Merck, Darmstadt, Germany) for 3 min. Primary antibodies were: AR 1:1000 (Abcam, ab9474), ERG 1:1000 (Santa Cruz, sc-354) and GAPDH 1:1000 (Abcam, ab8245).

### Immunofluorescence

Cells were plated in 8 compartment plates Millicell EZSlide (Millipore, Massachusetts, USA) at 1x10^5^ cells per well. After treatments, EZ slides were incubated with AR primary antibody (ab9474) overnight. Then, secondary antibody coupled to ALEXA fluorophore was added to the plate. Three different fields at 40 X were taken for each treatment with a confocal microscope.

### Cell viability

Cells were plated in 96-well plates at 1 × 10^4^ cells per well. 48 h after treatment for LNCaP cells and 96 h after treatment for VCaP cells, 10 μl of MTT reagent (SIGMA) were added per well, and cells were incubated at 37 °C and 5% of CO2 for 2 h. Next, medium was removed and 100 µl of DMSO (Applichem, Darmstadt) were added to solubilize formazan crystals in LNCaP cells. In VCaP cells, RPMI medium was not removed after treatment from well plates. We added 100 µl of SDS (10%) per well to solubilize formazan crystals overnight. Absorbance was read at 575 nm wavelengths in both cell lines.

### Cytometry assays

1 × 10^6^ cells were plated in 25 cm^2^ angled-flasks, except VCaP cells (1.5 × 10^6^ cells). 48 and 72 h after treatments, cells were trypsinized. We performed the assay with Cell cycle test kit (BD Biosciences, California USA) according to manufacturer’s instructions. Nocodazole (Sigma, St. Louis, MO, USA) at 1 μM was used to arrest the cell cycle in G2 phase. We used half of the cell population fated to apoptosis assays. In this case, cells were processed using Annexin V- FITC Kit (Thermo Fisher Scientific, V13242) according to manufacturer’s instructions. 2-amino-*N*-quinoline-8-yl-benzenesulfonamide (QBS) (SIGMA) was selected as an apoptosis inducer. Attune (Applied Biosystems, Life Technologies, Carlsbad, CA) was used for performing cell cycle and apoptosis assays.

### Wound healing

Wound healing assays were performed on 1 × 10^6^ cells per well in 6 well plates pre-treated with poly-l-Lisin. When cells reached confluence, a scratch was made in every well with a 10 μl pipette tip. Next, we treated cells, and wound closure was monitored every 24 h. Photos were taken and image analysis was performed using Image J software (version 1.52j).

### Invasion

VCaP cells (40,000) were seeded with 150 μl of DMEM medium and 10% FBS. Previously, calibration was performed on the plate with 30 μl of DMEM and FBS for 30 min. 20 microliters of a matrix (matrigel) were added in a 1:1 ratio for 30 min until polymerized. An initial reading was made to establish the background signal, and then the cell seeding was carried out 2 h before starting the reading to allow adherence, and then treatments were begun. The readings were made in real time on the cell culture every 15 min for 72 h using xCELLigence RTA (ACEA Biosciences, San Diego, California, USA).

### Xenograft murine model (VCaP)

3.5 × 10^5^ VCaP cells were inoculated into the back of twelve BALB/c nu/nu mice (4–6 weeks of age). Blood was collected to measure PSA by ELISA assay. When nude mice developed subcutaneous xenograft tumors at 250 mm^3^, then were divided into two treatment groups (3 mice in each group): Vehicle and SFRP1 after, castration surgery was performed. One week after castration, mice were randomized into two groups: control group (treated with PBS as vehicle) and problem group (treated with 50 µl of SFRP1 0.01 nM). SFRP1 protein and vehicle were subcutaneously administered around the tumor tissue once a week during 10 weeks. Mice were then euthanized, and tumor tissues were collected for mRNA, protein and IHC analysis. All animal procedures were performed according to NIH Animal Use and Care Guidelines (USA), and the local institutional committee: Comité Interno de Cuidados de Animales de Laboratorio of the Instituto Nacional de Cancerología (CICUAL-INCan) concerning the ethical use of experimental animals.

### Databases analysis

Gene expression profiling interactive analysis was used to identify the SFPR1 profile expression between normal tissues and prostate cancer [28]. cBioPortal was employed to determinate potential correlation between ERG and SFPR1 expression levels in prostate cancer tumors [29, 30].

### PSA quantitation by ELISA

Blood from the tail was collected by rubbing and dripping until obtaining an approximate volume of 400 μl. The collection was performed in yellow microtainer tubes (BD, New Jersey, USA). Once the serum was obtained, it was stored at − 70 °C for a few weeks until all of the samples were collected. The PSA ELISA kit (ABNOVA, China) was used in ELISA system, following the protocol established by the supplier.

### Immunohistochemistry (IHC)

Sections were dewaxed at 65 °C (paraffin melting temperature) for 15 min, followed by two passages through xylol for 15 min, 100% ethanol, (twice), and 70% ethanol for 5 min. Subsequently, tissues were rehydrated by two washes with PBS 1X for 5 min. Tissues were incubated in citrate buffer for 10 min in a pressure cooker for antigen recovery. Tissues were allowed to cool for 15 min, and then endogenous peroxidase was blocked with the peroxidase blocking solution (Bio BS) in a humid chamber (twice) for 30 min. Tissues were washed with 1X PBS, and incubated with the general blocking solution (Bio BS) in a humid chamber for 40 min. Tissues were then incubated with the corresponding primary antibodies at 4 °C in a humid chamber overnight. Afterwards, tissues were washed with 1X PBS to remove the excess primary antibody, and incubated with the secondary antibody and then with the polymer (MACH 3 Rabbit HRP Polymer Detection, BIOCARE) in a humid chamber for 10 min. Tissues were washed with 1X PBS to remove excess secondary antibody. The tissues were incubated with diaminobenzidine (DAB) diluted in diaminobenzidine buffer (1:100) and protein detection was carried out. Once the signal was detected, the reaction was stopped with PBS 1X. Tissues were counterstained with hematoxylin and allowed to dry. The slides were mounted with permount mounting medium to be visualized in the microscope (Olympus BX51). For IHC assays ERG was evaluated with anti-ERG (SANTACRUZ, sc-354) and Ki-67 with anti-Ki-67 (ABCAM, ab16655).

### Statistical analysis

The data were analyzed and plotted in GraphPad Prism 5 software (GraphPad Software, Version 5.01, La Jolla, CA, USA). Analysis of PCR data was performed using the 2^(−delta delta CT) method. The statistical analysis of the relative gene expression levels was performed by one-way ANOVA followed by a Tukey post hoc test. Values of p < 0.05 were considered statistically significant.

## Results

### SFRP1 expression in non-malignant and prostate cancer cells

**Fig. 1:**
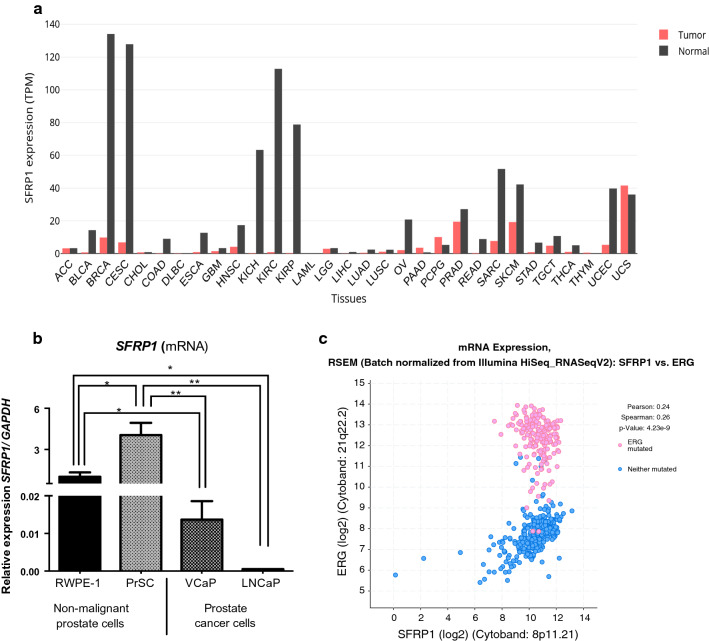
SFRP1 expression in non-malignant and prostate cancer cells. **a** SFRP1 expression level is decreased in prostate cancer tumor (red), but increased in normal tissues (black). **b** Differential expression of SFRP1 in different prostate cell lines. SFRP1 expression in VCaP cells (positive to TMPRSS2-ERG gene fusion) was higher than in LNCaP (fusion negative) cells under basal conditions, *p ≤ 0.0122, **p ≤ 0.0019. **c** Co-expression analysis of ERG and SFRP1 in 494 samples of prostate adenocarcinomas obtained from cBioPortal platform

First, SFRP1 profile expression between tumor and normal prostate tissues was obtained using GEPIA database. SFRP1 up levels was found in normal prostate tissues but down levels in prostate cancer samples (Fig. 1a). Later, the SFRP1 mRNA expression was investigated in non-malignant prostate cells and prostate cancer cells (Fig. 1b). The latter expressed lower levels of SFRP1 compared with normal prostate cells: RWPE-1 (epithelial) and PrSC (stromal). However, differential expression was observed between VCaP cells (positive for TMPRSS2-ERG) and LNCaP cells (negative for TMPRSS2-ERG). Although both cell lines have low SFRP1 expression, VCaP cells presented higher SFRP1 expression compared to LNCaP cells (Fig. 1b). We performed a co-expression analyses of SFRP1 and ERG in 494 prostate adenocarcinomas samples from the TCGA database [31]; as expected, low positive correlation between SFRP1 and ERG expression was confirmed (Spearman (0, 26) and Pearson (0, 24) (Fig. 1c). Only 1.8% (9 biopsies) presented SFRP1 amplification, and 3.5% exhibited a SFRP1 deletion opening the door to evaluate the effect of exogenous SFRP1.

### Effect of exogenous SFRP1 protein on AR’s transcriptional activity

**Fig. 2:**
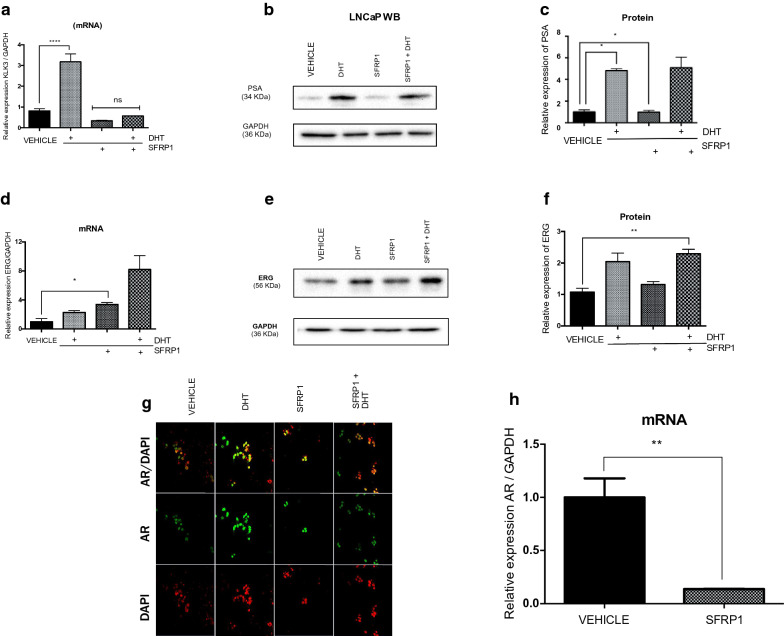
Effect of SFRP1 on AR target gene expression. KLK3 (PSA) expression after SFRP1 treatment, ****p ≤ 0.0001, *p ≤ 0.01, (**a**–**c**). Effect of exogenous SFRP1 on ERG expression *p ≤ 0.05, **p ≤ 0.005 (**d**–**f**). AR localization by Immunofluorescence. Red (DAPI) and green (AR), (**g**). Expression of AR, **p ≤ 0.01 (**h**). Cells were treated with DHT and SFRP1 for 24 h (0.01 nM)

To determine whether exogenous SFRP1 protein could have some effect over PCa cells positive to TMPRSS2-ERG, we performed assays to measure indirectly AR transcriptional activity through genes with ARES sequences. We treated cells with 5α-Dihydrotestosterone (DHT) as positive control and exogenous SFRP1 protein was the recombinant protein. The concentration of SFRP1 was chosen respect to DHT had a significant increase in cell viability to resemble the physiological context where the lowest concentrations of protein could counteract or enhance the effect of the hormone (Additional file 1: Figure S1). Then, the expression of androgen responsive genes *KLK3* (gene coding to PSA) and ERG (functional product of TMPRSS2-ERG fusion) was evaluated in LNCaP and VCaP cells, respectively. In LNCaP cells, SFRP1 treatment did not modify *KLK3* expression at mRNA and protein but in combination with DHT, SFRP1 seems to decrease the effect of DHT (Fig. 2a–c). Interestingly, in VCaP cells; contrary to LNCaP, the addition of SFRP1 protein increased the expression of ERG (Fig. 2d–f). In order to determine if SFRP1 promotes ERG expression via AR translocation, immunofluorescence assays were performed and we observed that SFRP1 promotes the translocation of AR to the cell nucleus in a similar way to DHT (Fig. 2g). To demonstrate if this effect is due by the AR’s translocation but not by the AR expression, we also determined AR expression after SFRP1 treatment, and found that SFRP1 decreased AR expression in VCaP cells as previous reports (Fig. 2h).

### Effect of SFRP1 over cell viability and cell cycle

**Fig. 3:**
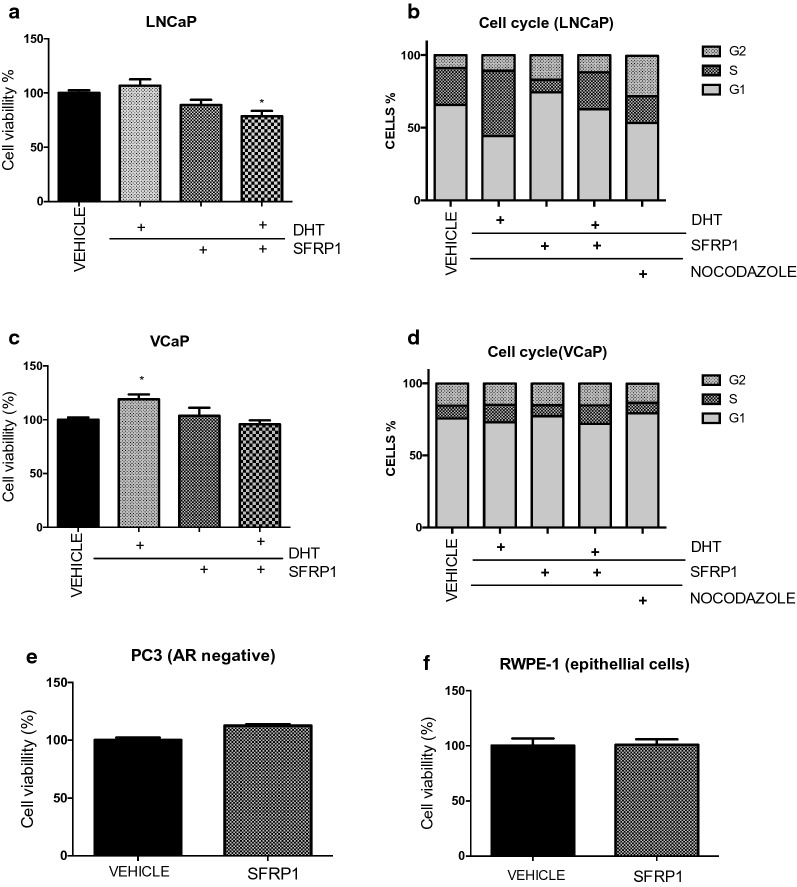
Effects of SFRP1 on cell viability and cell cycle. Cells were treated with DHT (0.01 nM) and SFRP1 (0.01 nM). Cell viability was measured by MTT assay and cell cycle was determined by flow cytometry with IP. **a** LNCaP cells, *p ≤ 0.01 vs vehicle (**a**–**d**). Effects of SFRP1 on cell viability in PC3 and RWPE-1 cells (**e**–**f**). In the cytometry assays, LNCaP, PC3 and RWPE-1 cells were treated for 48 h, and VCaP for 72 h. Nocodazole was used as G2 arrester at 1 μM

In LNCaP cells, SFRP1 treatment decreased cell viability to 88.9% and when it was co-treated with DHT, cell viability diminished 22% (Fig. 3a). In line with that, SFRP1 decreased the number of cells in S phase from 25.6 to 8.8% and increased cells in G1 phase from 65.5 to 74.2% (Fig. 3b and Table 1). However in VCaP cells, we did not observe significant changes neither cell viability nor cell cycle after SFRP1 treatment (Figs. 3c, d and Table 2). We also ask if SFRP1 could protect cells for apoptosis by FACS assay however we don’t observe significant changes compared to vehicle (Additional file 1: Figure S2). Hence, we performed a cell viability test to investigate the potential effect of SFRP1 on PCa cells that did not express AR or TMPRSS2-ERG but SFRP1 and how was expected, not effect was observe in PC3 (AR negative) and RWPE-1 (non-cancerous epithelial cells (Fig. 3e, f). This suggests that SFRP1 it could be affecting another type of neoplastic feature as migration in VCaP cells positive to AR and TMPRSS2-ERG.

**Table 1:** Cell cycle in VCaP cells

Cell cycle phase	Vehicle	DHT	SFRP1	SFRP1 + DHT	Nocodazole
G1	65.5% ± 3.8	45.1% ± 8.5**	74.2% ± 0.47	62.6% ± 2.8	53.6% ± 1.69
S	25.6% ± 2.6	45.1% ± 11.5*	8.8% ± 0.81	25.4% ± 2.4	18.8% ± 1.35
G2	8.9% ± 1.7	10.7% ± 3.05	17% ± 0.45	12% ± 0.60	27.6% ± 0.23*

Data are the average ± s.d. of a representative experiment carried out in triplicate (*p < 0.005; Student’s t-test of treatments compared to vehicle)

**Table 2:** Cell cycle in LNCaP cells

Cell cycle phase	Control	DHT	SFRP1	SFRP1 + DHT	Nocodazole
G1	75.6% ± 0.2	73.05% ± 2.2	77.21% ± 0.5	71.95% ± 3.1	79.31% ± 0.2
S	9.0% ± 0.1	12.04% ± 3.2	7.66% ± 0.3	12.76% ± 2.4	7.36% ± 0.2
G2	15.3% ± 0.3	14.91% ± 1.1	15.12% ± 0.1	15.29% ± 1.6	13.34% ± 0.07

Data are the average ± s.d. of a representative experiment carried out in triplicate (*p < 0.005; Student’s t-test, of treatments compared to control (vehicle)

### Effect of SFRP1 over cell migration and invasion

**Fig. 4:**
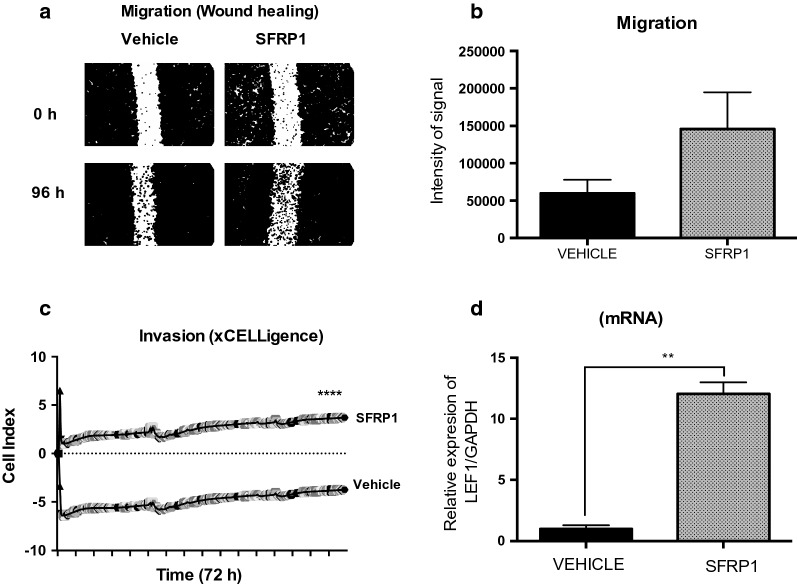
Effects of SFRP1 on cell migration and invasion. Cell migration by wound healing assay, VCaP cells were treated for 96 h, increased migration was observed but was not significant in this assay (**a**, **b**). Cell invasion analyzed by xCELLigence system, VCaP cells were treated for 72 h and invasion was monitored in real-time every 15 min, ****p ≤ 0.0001 (**c**). Expression of LEF-1 in VCaP cells, **p ≤ 0.0045 (**d**). Cells were treated for 24 h with DHT and SFRP1 (0.01 nM each)

We decided continue assessing if SFRP1 could have an effect over migration in VCaP cells (positive to TMPRSS2-ERG) by measuring wound healing during 96 h. Significant increase in cell migration was observed in cells treated with SFRP1 compared to vehicle (Fig. 4a, b). To evaluate if the effect of SFRP1 protein over cell migration could promote invasion, we used sofisticated xCELLigence system. We found that SFRP1 also increases VCaP cell invasion. We followed invasion in real-time every 15 min for 72 h, increased invasion was observed from the first hours of the experiment (Fig. 4c). Furthermore, SFRP1 augmented tenfold the expression of the metastatic-related gene LEF-1 that belong to WNT pathway and its related to the EMT (Fig. 4d). Finally, we performed W.B. to evaluate two EMT markers and a tendency of increased N-cadherin and decreased E-cadherin was observe after 24 h of SFRP1 treatment (Additional file 1: Figure S3).

### Effect of SFRP1 on VCaP xenograft

**Fig. 5:**
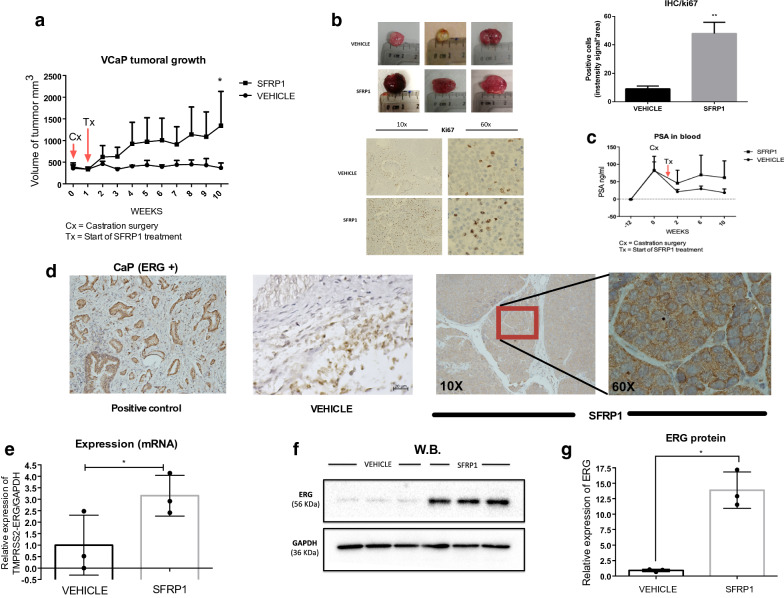
Effect of SFRP1 protein in VCaP xenograft. SFRP1 treatment was performed every week for 10 weeks. SFRP1 was subcutaneously applied around the tumor (50 µl at 0.01 nM) (**a**). Representative photography for every xenograft in each treatment. In IHC, Ki67 as proliferative marker were showed at ×10 and ×60 magnification. ki67 IHC at 10X was quantify as signal of positive cells*area, **p ≤ 0.008 (**b**). Human PSA was quantified by ELISA (**c**). Immunohistochemistry of VCaP xenografts, tissues were collected after euthanasia in formalin, then embedded in paraffin, and IHC for ERG was performed (**d**). *TMPRSS2*-*ERG* expression (mRNA) and ERG (protein) in VCaP xenografts. Tissue was processed for RNA isolation and protein extraction and RT-qPCR and W.B. were performed, *p ≤ 0.03 (mRNA) and *p ≤ 0.01 (protein) (**e**–**g**)

We performed a pilot test using a castrated murine xenograft model made with VCaP cells in order to investigate the effect of SFRP1 in vivo without hormone. We inoculated VCaP cells subcutaneously and when xenografts reached near to 300 mm^3^ testicles of mice were removed. Then, we started the treatment with SFRP1 administered subcutaneously surrounding the tumor once per week for 10 weeks. We observed that SFRP1 protein promoted the growth of xenografts up to 1000 mm^3^. Furthermore, in xenografts treated with SFRP1 protein significant changes were observe for Ki67, a proliferation’s marker commonly used in IHC. Both, quantitation of signal and IRS (immunoreactivity score) (Additional file 1: Figure S4) were calculated as described by Parashar et al. and Fedchenko et al. (Fig. 5a, b) [32, 33]. Additionally, during the 10 weeks of treatment, blood was collected and human PSA levels from the tumor were quantified. Only a tendency towards higher PSA levels was observed in the mice treated with SFRP1 protein but no significant differences were found between groups (PSA in untreated group: 18.1 ng/ml ± 11.04 vs PSA in treated group: 61.6 ± 48.5) (Fig. 5c). After treatment, mice were euthanized, and xenograft tissue collected to measure ERG by IHC. Higher amounts of ERG protein were observed in the tumors of animals that received SFRP1 (Fig. 5d). There was also a significant increase of ERG after SFRP1 treatment at the mRNA and protein levels (Fig. 5e–g).

## Discussion

The aim of this work was to evaluate the effect of exogenous SFRP1 protein on PCa cells expressing TMPRSS2-ERG fusion. SFRP1 protein belongs to the WNT signaling pathway and regulates signaling together with other WNT-related proteins such as WNT3a, WIF-1 and Cerberus [34–37]. As far as we know, there are no works that evaluates the role of SFRP1 protein in PCa positive to TMPSS2-ERG. Previous reports have shown that SFRP1 is expressed at low level in PCa tumors and cell lines, however there are no reports in models of PCa with TMPRSS2-ERG fusion [22, 38]. First, we performed in silico screening using databases and found SFRP1 is down-expressed in tumor samples with poor positive correlation between the expression of SFRP1 and ERG in PCa samples as expected. In addition, we observed that the expression of SFRP1 is low in PCa cells compared to elevated expression in non-malignant prostatic cells from stroma and epithelium. This is in line with previous reports, however these reports did not take into account the presence of TMPRSS2-ERG [22, 39–41]. Importantly, previous reports have demonstrated that SFRP1 protein could be an important modulator of stromal–epithelial communication in PCa progression [26, 42]. Recent reports show that SFRP1 it could have biphasic effects depending on concentration, taking account that, we performed assays with low concentrations of SFRP1 to resemble exogenous protein secreted from stroma [43, 44].

In LNCaP and 22RV1 cells, SFRP1 was reported as negative regulator of AR’s transcriptional activity [20] and we observed the same effect in our study with LNCaP cells when the expression of *KLK3* (mRNA) and PSA (protein) were measured. In VCaP cells (positive to TMPRSS2-ERG), contrary to LNCaP, SFRP1 promoted the expression of ERG and the translocation of AR to the cell nucleus was observed. In addition, using immunofluorescence and RT-qPCR we corroborated that the effect of SFRP1 depends on AR translocation but not over its expression, this is in line with previous reports where it’s observed that ERG decrease the expression of AR [19]. These data indicate that AR increases its transcriptional activity in the presence of SFRP1, activating androgen-responsive genes such as ERG in VCaP cells. It is not clear why SFRP1 protein have the opposite effects between LNCaP and VCaP cells but this could explain in some manner the diverse responses that exist in different cell types associated with tumor heterogeneity. For example, reports show that AR of LNCaP and VCaP share near to 61% of the AR-bound regions in the VCaP cells overlapped and co-occupancy of ERG in this sites showing differences in the activity of AR between these cells [15, 19, 20, 41, 42, 45–47]. Furthermore, LNCaP cells are PCa cells negative for the *TMPRSS2*-*ERG* fusion and represent a stage of disease that is not resistant to castration; LNCaP cells were isolated from a metastatic lesion to the lymph node and the patient did not receive androgen blockade therapy [48–52]. Conversely, VCaP cells are positive for the TMPRSS2-ERG fusion, and they were isolated from a metastatic lesion to vertebral bone and the patient did receive androgen-blockade therapy perhaps representing a more advanced state of the disease [53].

**Fig. 6:**
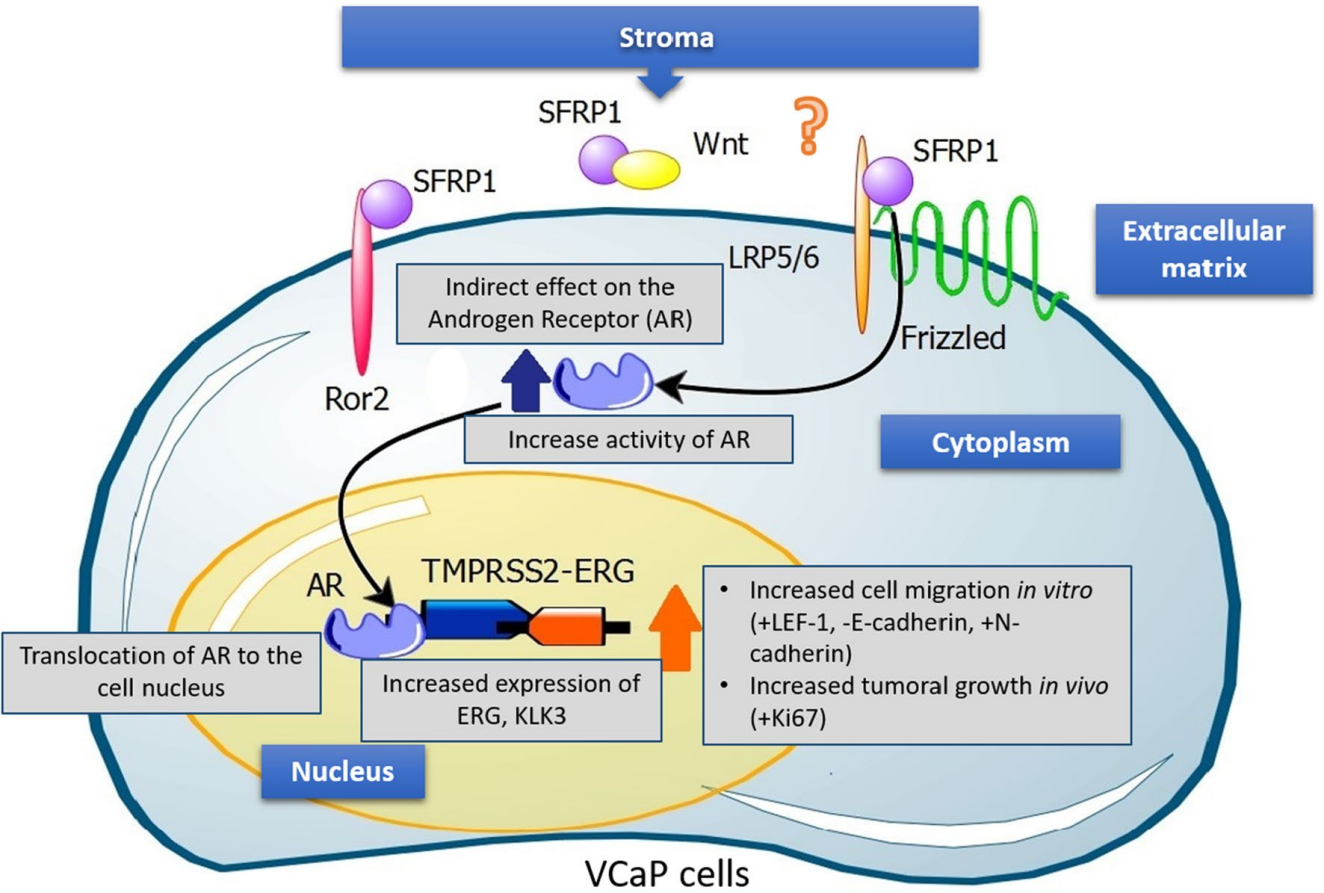
Possible mechanisms about of SFRP1 effect on VCaP cells. Exogenous SFRP1 protein induce indirect effect on the transcriptional activity of Androgen Receptor promoting cell migration in vitro and cell growth in vivo

We performed cell viability and cell cycle assays to evaluate the functional impact of exogenous SFRP1 on PCa cells. In LNCaP cells, SFRP1 decreased the S phase and increases the G1 phase of the cell cycle, however in VCaP cells, we did not observe significant differences in either viability or cell cycle. It is important to note that is reported that VCaP cells have a low rate of duplication, and ERG has been mostly associated with cell migration and invasion [17, 24]. Thus, we observed that SFRP1 protein promoted both migration and cellular invasion by wound healing assay and sophisticated xCELLigance system. The main advantage of this system is that was specially designed with microtiter plates containing interdigitated gold microelectrodes to noninvasively monitor cultured cells using electrical impedance as the readout allowing accurate readings from the first minutes. Since LEF-1 has been related to tumor progression to metastasis and this gene has been reported to be regulated for ERG, we measured it’s expression in VCaP cells after treatment with exogenous SFRP1 [17, 24, 54]. Treatment promoted the expression of LEF-1 a gene involved in the end of WNT cascade signaling, however this needs further investigation to establish if complete activation of WNT signaling it´s happening with SFRP1 in cells positive to TMPRSS2-ERG. Finally, in order to determine the effect of SFRP1 protein on PCa cells in vivo, we performed a pilot murine xenograft model using VCaP cells. There are only a few studies of xenografts established from VCaP cells in murine models and few that evaluate the effect of SFRP1 in the absence of androgens [52]. We performed castration surgery when the tumor reached 300 mm^3^ and after 10 weeks of treatment with subcutaneously SFRP1 per week. We observed that SFRP1 protein increased tumors in size by proliferation measuring Ki-67, also a tendency to high levels of human PSA in mice serum was observed. Finally, xenograft tissues also showed a significant increase in ERG expression at both mRNA and protein levels by IHC, RT-qPCR and WB. In this study we added a possible mechanism which exogenous SFRP1 protein could induce its effect in VCaP cells. Previously reports has described, how SFRP1 can interact with receptors as Frizzled, LRP5/6, Ror; interact with WNT’s proteins and even others SFRP’s. However, in this case further investigation is needed to clarify exactly what receptors could be binding of SFRP1 in VCaP cells (Fig. 6) [21, 55].

## Conclusions

SFRP1 protein showed divergent effects between LNCaP (negative to TMPRSS2-ERG) and VCaP (positive to TMPRSS2-ERG) cells. In VCaP cells, SFRP1 promoted AR’s transcriptional activity and increased TMPRSS2-ERG expression. This effect increased migration and invasion in vitro and the growth of a tumor xenograft in vivo. In PCa patients that received androgen-blockade therapy after 18–36 months usually show an increase in PSA levels in the absence of androgens, and metastasis occurs [56]. This could be explained by the participation of exogenous molecules from stroma such as SFRP1 that could be activating AR signaling in TMPRSS2-ERG tumors. Although it has been reported that 50% of PCa tumors express the TMPRSS2-ERG fusion, few tests are currently performed on patients to determine the expression of this fusion. The interplay between paracrine molecules and AR activity could have clinical implications in absence of androgens and could be drive to new drug design [57, 58]. We consider that in the future, the evaluation of the co-expression of SFRP1 (stroma) and TMPRSS2-ERG (epithelium) in PCa tumors could help to improve the prognosis and it could make better decisions about pharmacological treatments. This is the first work that evaluates the role of exogenous SFRP1 protein on VCaP cells; however, more experiments will be necessary to establish completely, downstream mechanisms of SFRP1 on PCa positive to TMPRSS2-ERG fusion.

## Supplementary information

**Additional file 1.Figures S1–S4. Figure S1**: Cell viability in LNCaP and VCaP cells treated with DHT hormone. **Figure S2**: Apoptosis plots of VCaP cells treated with SFRP1. **Figure S3**: Western Blot of E-cadherin and N-cadherin in VCaP cells treated with SFRP1. **Figure S4**: Immunoreactive score of IHC signal for Ki67 proliferation’s marker from VCaP xenografts treated with SFRP1.

## Data Availability

The datasets during and/or analyzed during the current study available from the corresponding author on reasonable request.

## Abbreviations

AR: Androgen receptor
PCa: Prostate cancer
*TMPRSS2*-*ERG*: Fusion gene that joins the transmembrane serin-protease TMPRSS2 (Transmembrane Serine Protease 2) with the transcriptional factor ERG (ETS-related gene)
SFRP1: Secreted frizzled-related protein of WNT signaling
