# Supplementary figures and images for: SFRP1 increases TMPRSS2-ERG expression promoting neoplastic features in prostate cancer in vitro and in vivo
Source: Cancer Cell Int. 2020 Jul 16;20:312. doi: 10.1186/s12935-020-01333-5 (PMC7364616; doi:10.1186/s12935-020-01333-5)

Figure S1

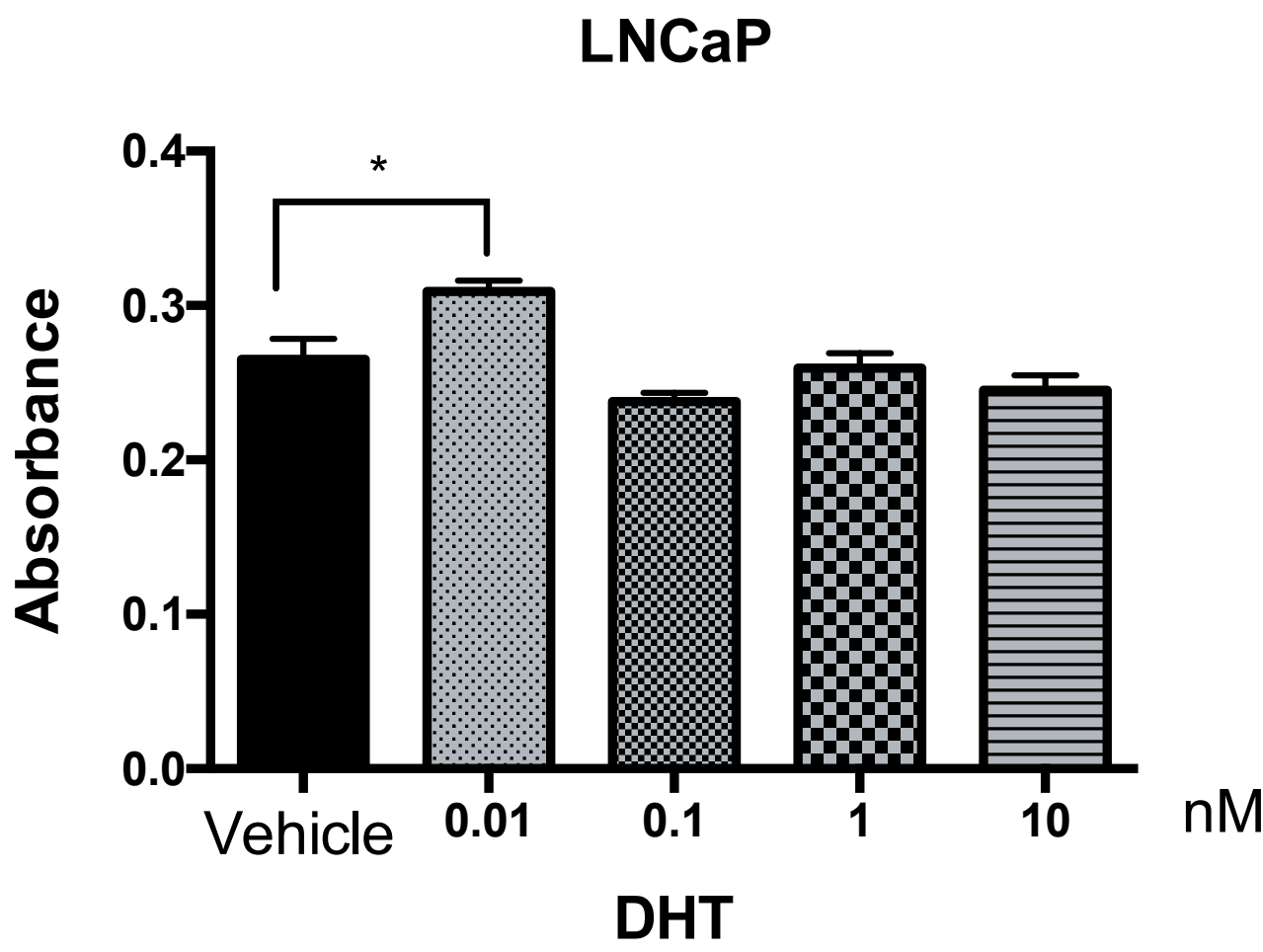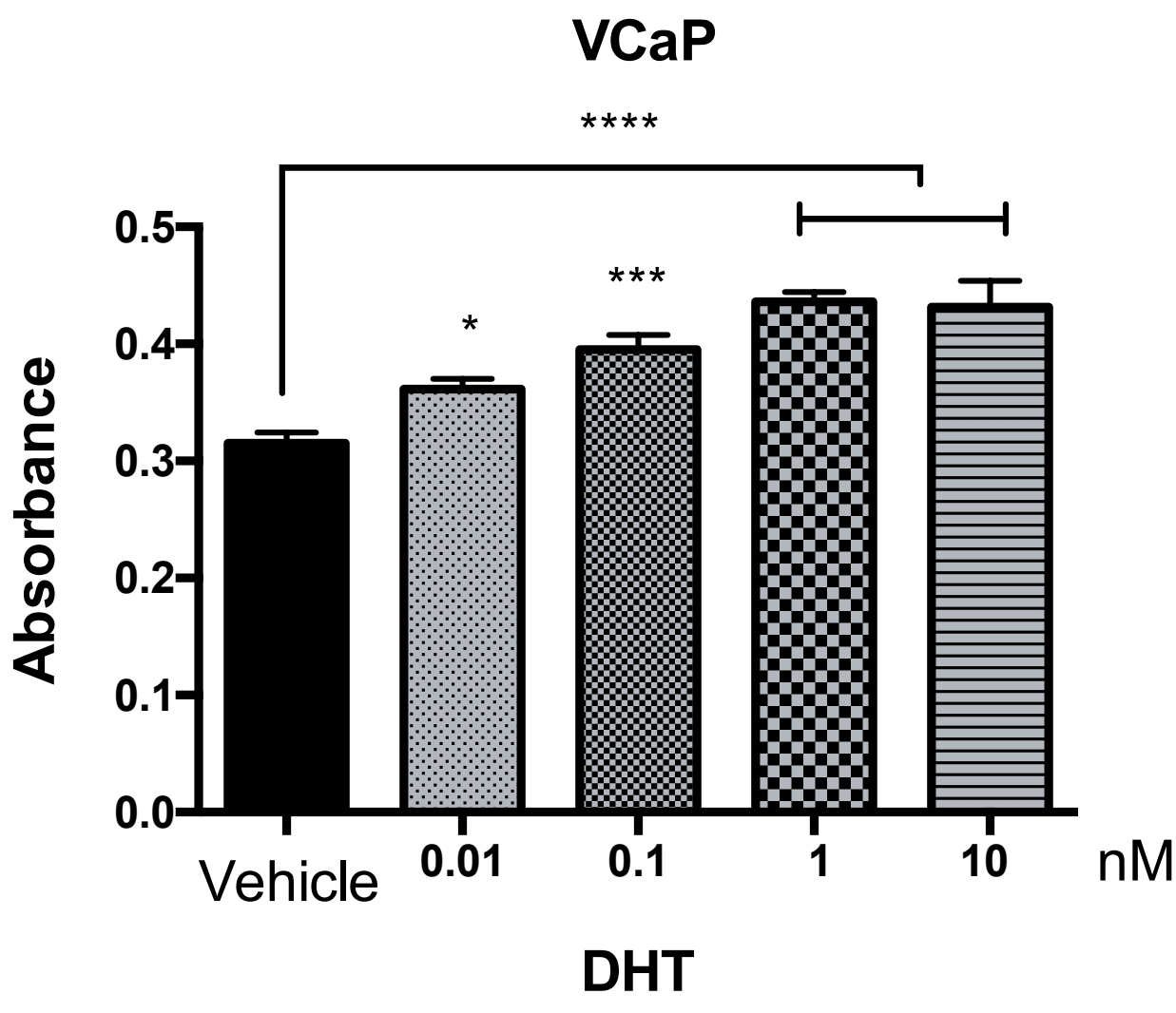

Figure S2

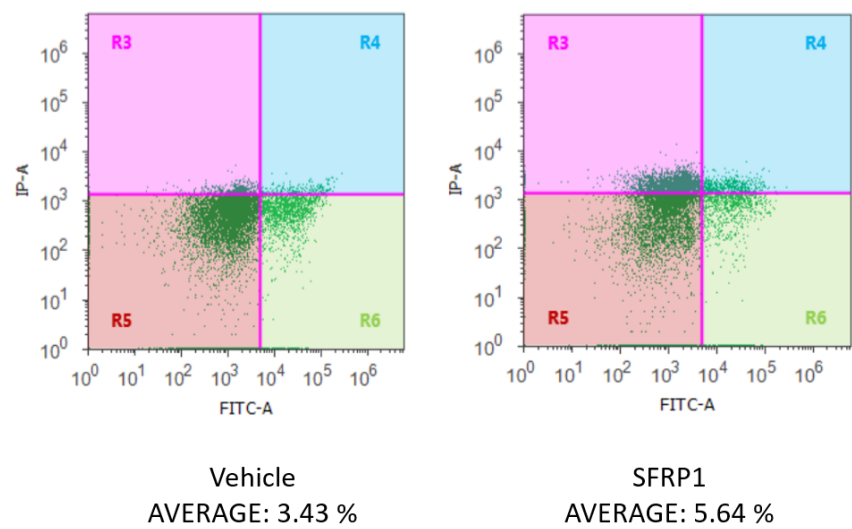

Figure S3

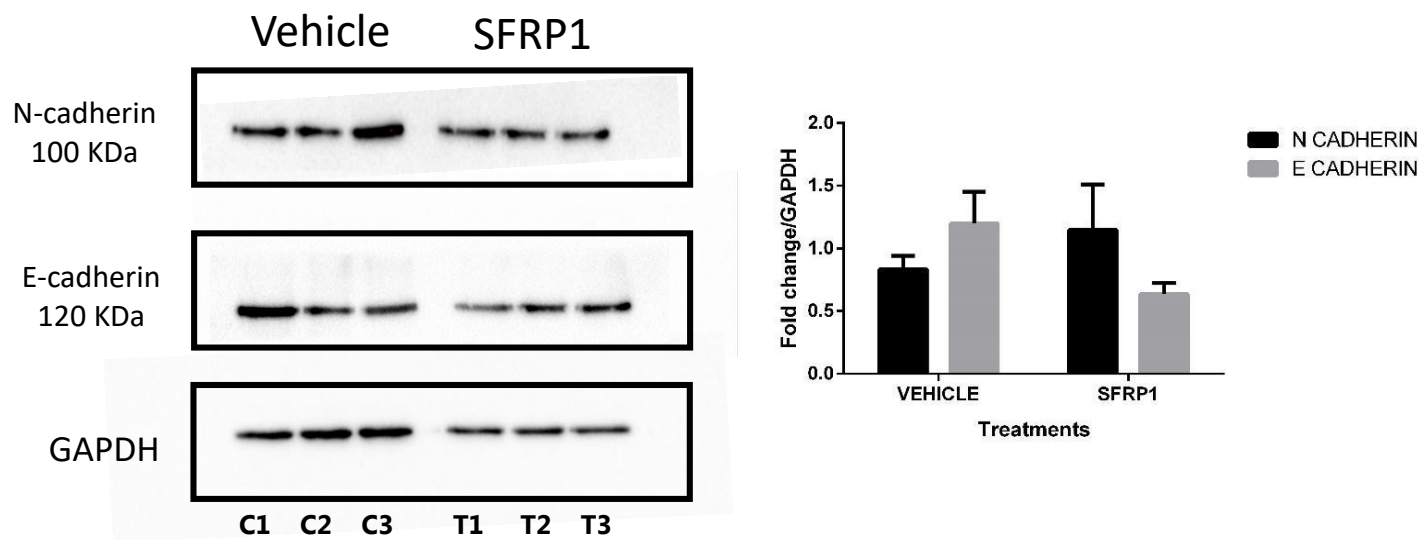

Figure S4

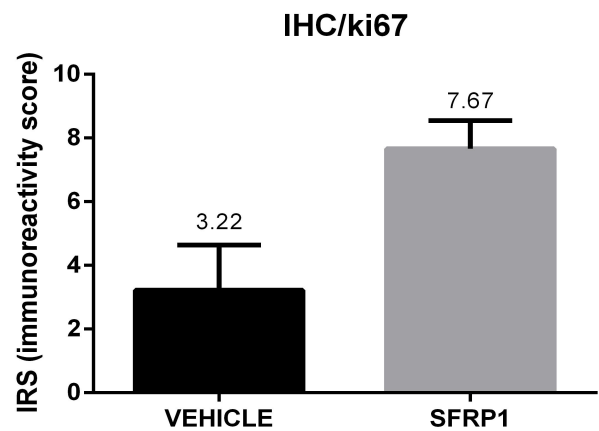

Supplement: Supplementary file 1 — Additional file 1.Figures S1–S4. Figure S1: Cell viability in LNCaP and VCaP cells treated with DHT hormone. Figure S2: Apoptosis plots of VCaP cells treated with SFRP1. Figure S3: Western Blot of E-cadherin and N-cadherin in VCaP cells treated with SFRP1. Figure S4: Immunoreactive score of IHC signal for Ki67 proliferation’s marker from VCaP xenografts treated with SFRP1. [file 12935_2020_1333_MOESM1_ESM.pdf]
